# Construction Notes

**Published:** 1893-12-16

**Authors:** 


### CONSTRUCTION NOTES.

### Isolation Hospital at Tamworth.

The erecting of this hospital has just been completed; tenders, last November, having been accepted from Mr. E. Williams, a local contractor, to build the same for £1,700. The buildings are arranged in three substantial blocks, one of which consists of the house for the superintendent and matron, containing also doctors' and nurses' sitting-rooms, four large bed-rooms, bath-room, &c. The hospital proper consists of the other two blocks, both of which have one storey only, each containing three lofty and well-ventilated apartments. There is in all accommodation for twelve beds. The floors are laid with Eber's patent pitch pine blocks, and Boyd's patent stoves are fitted up in each ward. There is also a mortuary and an outside kitchen. The drainage is by means of dumb wells, with gravelly bottoms.

### Devon and Cornwall Homœopathic Hospital.

Considerable alteration has transformed a private residence into this well-arranged and substantial hospital, and much credit is due to the architects, Messrs. King and Lester, and to the contractor, Mr. W. H. Lethbridge, on the success of the work. It is so arranged that the basement floor provides kitchen, scullery, pantries, larder, and nurses' dining-room; whilst the ground floor consists of the board-room, men's ward for six beds, and nurse's sitting-room; the first floor a women's ward for six beds, children's ward, and bath-room; the second floor two private wards for paying patients, matron's room, and operating-room; and the third floor consists of four bed-rooms for nurses and servants. The stable buildings at the rear have been converted into a dispensary, with a separate entrance, which also gives access to an operating, a waiting, and a doctor's room.

